# Preserved Milk

**Published:** 1906-09

**Authors:** 


PRESERVED MILK.—The exposures of the dairymen in this part of Texas by *Holland's Magazine* for September, has thoroughly aroused the people and the cities have promptly put a stop to the use of formaldehyde or "milk sweet." The Houston, San Antonio, Dallas, and Austin papers have done excellent missionary work. This is one of the most hopeful signs of the times. The lay press has done what the medical press could never do: enlightened the masses and warned them. We now have strong hopes that the Thirtieth Legislature will give us a law to prevent adulteration: a pure food law. The bill passed by Congress only protects a State from introduction of adulterated articles from outside. Let our Legislative Committee "make a specialty" of a pure food bill.

